# Using data envelopment analysis to perform benchmarking in intensive care units

**DOI:** 10.1371/journal.pone.0260025

**Published:** 2021-11-18

**Authors:** Bianca B. P. Antunes, Leonardo S. L. Bastos, Silvio Hamacher, Fernando A. Bozza

**Affiliations:** 1 Department of Industrial Engineering, Pontifical Catholic University of Rio de Janeiro (PUC-Rio), Rio de Janeiro, RJ, Brazil; 2 Oswaldo Cruz Foundation (FIOCRUZ), Rio de Janeiro, RJ, Brazil; 3 D’Or Institute for Research and Education (IDOR), Rio de Janeiro, RJ, Brazil; 4 Brazilian Research in Intensive Care Network (BRICNet), São Paulo, Brazil; San Giuseppe Hospital, ITALY

## Abstract

**Background:**

Studies using Data Envelopment Analysis to benchmark Intensive Care Units (ICUs) are scarce. Previous studies have focused on comparing efficiency using only performance metrics, without accounting for resources. Hence, we aimed to perform a benchmarking analysis of ICUs using data envelopment analysis.

**Methods:**

We performed a retrospective analysis on observational data of patients admitted to ICUs in Brazil (ORCHESTRA Study). The outputs in our data envelopment analysis model were the performance metrics: Standardized Mortality Ratio (SMR) and Standardized Resource Use (SRU); whereas the inputs consisted of three groups of variables that represented staffing patterns, structure, and strain, thus resulting in three models. We compared efficient and non-efficient units for each model. In addition, we compared our results to the efficiency matrix method and presented targets to each non-efficient unit.

**Results:**

We performed benchmarking in 93 ICUs and 129,680 patients. The median age was 64 years old, and mortality was 12%. Median SMR was 1.00 [interquartile range (IQR): 0.79–1.21] and SRU was 1.15 [IQR: 0.95–1.56]. Efficient units presented lower median physicians per bed ratio (1.44 [IQR: 1.18–1.88] vs. 1.7 [IQR: 1.36–2.00]) and nursing workload (168 hours [IQR: 168–291] vs 396 hours [IQR: 336–672]) but higher nurses per bed ratio (2.02 [1.16–2.48] vs. 1.71 [1.43–2.36]) compared to non-efficient units. Units from for-profit hospitals and specialized ICUs presented the best efficiency scores. Our results were mostly in line with the efficiency matrix method: the efficiency units in our models were mostly in the “most efficient” quadrant.

**Conclusion:**

Data envelopment analysis provides managers the information needed to identify not only the outcomes to be achieved but what are the levels of resources needed to provide efficient care. Different perspectives can be achieved depending on the chosen variables. Its use jointly with the efficiency matrix can provide deeper understanding of ICU performance and efficiency.

## 1 Introduction

Benchmarking is essential to identify opportunities for improvement in healthcare systems, especially for high cost and complex services as Intensive Care Units (ICUs) [1]. Several metrics have been proposed to evaluate ICU performance, mainly the risk-adjusted mortality and resource use [2, 3].

Data Envelopment Analysis (DEA) is a method based on frontier analysis that compares the efficiency among units, widely used in business benchmarking [4]. Other methodologies frequently use single or combined metrics in regression analysis [5, 6] to benchmark units and identify organizational characteristics. Compared to those studies, DEA has the advantage of comparing the relative performance of units individually instead of estimating the average efficiencies among groups of units [7]. DEA models are nonparametric and convene multiple inputs (e.g., organizational characteristics, staffing, availability of resources) and outputs (e.g., performance metrics) in a single model, which is especially important to the healthcare environment [8] and can lead to more accurate results [9].

Applications of DEA in healthcare are still scarce and have focused on analyzing the performance of hospitals [10, 11], departments [12], or even countries [13] regarding the number of inpatients/discharges [14, 15], days of treatment [16], and costs/revenue [14]. In the context of ICUs, DEA studies have analyzed the performance of nurses [17] or patients’ conditions [7], but only a few have used it to effectively compare units [14–16, 18–20].

Our study aimed to perform a benchmarking analysis of ICUs using a DEA model. We used data from the ORCHESTRA study [21, 22], a large multicenter dataset of Brazilian ICUs. We considered organizational characteristics as the main inputs and risk-adjusted mortality and resource use metrics as the outputs. We compared the DEA results to the efficiency matrix [3, 23], which is a classical approach often used for ICU benchmarking.

## 2 Materials and methods

### 2.1 Data source, study design, and population

We performed a retrospective analysis on observational data of patients admitted to ICUs in Brazil and considered for the ORCHESTRA study [21]. Local Ethics Committees at the D’Or Institute for Research and Education (IDOR, Parecer: 334.835) and the Brazilian National Ethics Committee (*Certificado de Apresentação para Apreciação Ética*—CAAE: 19687113.8.1001.5249) approved the data collection and the need for informed consent was waived. Data comprised of routinely collected anonymized information from ICU-admitted patients was routinely collected data for administrative use from an electronic system used for benchmarking purposes (Epimed Monitor®, Rio de Janeiro, Brazil) [24]. Detailed information on data collection, centers, and participant selection was previously published [21, 22]. Briefly, we considered adult patients (≥ 16 years old) admitted to the ICUs from January 2014 to December 2015. Readmissions and patients with missing core data (such as age, main diagnosis, and length of stay) were excluded [21]. The database contains information in three levels: patients (demographic data, severity of illness at admission, and outcomes), hospitals (e.g., infrastructure, type, accreditation), and ICUs (infrastructure, staffing patterns, and organizational aspects).

### 2.2 Data Envelopment Analysis (DEA)

In a DEA model, the efficiency of a Decision-Making Unit (DMU) is calculated as the ratio of the weighted sum of outputs (outcomes) to the weighted sum of inputs (resources). Those weights are defined by maximizing the efficiency of a DMU, with the constraint that each DMU cannot have an efficiency greater than 1 [25]. The model calculates the efficiency of each DMU using the others as targets, resulting in the relative efficiency of that unit. Therefore, to obtain all units’ efficiency scores, it is necessary to run the model for all units. A DMU is considered "efficient" if its relative efficiency equals one and, therefore, it can be considered a reference to others. In our model, we considered each ICU in our dataset as a DMU.

#### 2.2.1 Inputs and outputs

As outputs in our DEA model, we considered two performance metrics: Standardized Mortality Ratio (SMR) and Standardized Resource Use (SRU). SMR is the ratio between observed and expected deaths in the ICU [2]. The expected deaths were estimated using the SAPS-3 (Simplified Acute Physiology Score 3) standard mortality equation [26]. SRU is the observed-to-expected use of resources: we considered the ICU length-of-stay (LOS) as a surrogate measure of resource utilization [3].

As inputs, we considered three groups of variables: staffing, structure, and strain. The staffing variables corresponded to the availability of ICU healthcare professionals (physicians, nurses, nursing technicians, and physiotherapists) per 10 ICU beds ratio. The structure variables are comprised of the number of ICU beds and the medical team shift scheduling (number of working hours per shift per week). As strain variables, we considered the bed-occupancy rate to indicate supply-demand mismatch in the ICU [27].

#### 2.2.2 Model specifications

We developed three DEA models with the outputs SMR and SRU and three groups of inputs (**Table 1**). We considered a Variable Returns to Scale (VRS) approach for all models since a change in inputs may not lead to a proportional change in outputs (a description of VRS DEA models is presented in the Supplementary Material). Also, the VRS is more suitable for models in which the units differ in size [15]. The idea under traditional DEA is that an increase in inputs generates an increase in outputs. However, as we considered undesirable outputs (the lower, the better), we used the inverse values (1/output) [28]. This was also the case for the bed occupancy rate input since low occupancy rates are easier to handle and may result in better outcomes.

**Table 1:** Specifications of the DEA models.

Model	Focus	Inputs	Outputs	Orientation	Research question
**A**	Staffing	• Number of physicians per 10 beds;	• SMR	Input	Given a certain performance in terms of SMR and SRU, how much lower should the staffing proportion be?
			• SRU		
		• Number of nurses per 10 beds;			
		• Number of nursing technicians per 10 beds;			
		• Number of physiotherapists per 10 beds.			
**B**	Structure	• Number of ICU beds;	• SMR	Output	Given a certain structure in terms of beds and volume of professionals, how much lower should the SMR and SRU be?
		• Total physicians’ hours per week;	• SRU		
		• Total nurses’ hours per week.			
**C**	Capacity	• Bed Occupancy Rate	• SMR	Input	Given a certain performance in terms of SMR and SRU, how much higher should the occupancy rate be?
			• SRU		

Model A provides the staffing perspective: the higher the proportion of medical professionals per 10 beds, the lower should the units’ SMR and SRU be. Therefore, units with high staffing proportion or high SMR and SRU compared to the others in the dataset may not be considered efficient. Model B compares the SMR and SRU of units with similar sizes, considering the number of beds and hours worked by the medical team. Model C compares the bed occupancy rate of units that had similar performance.

DEA models have pre-specified orientations: in input-oriented models, the efficiency is defined by reducing the inputs and holding the outputs constant, whereas the output-oriented models consider the opposite [28]. We defined models A and C as input-oriented because the inputs are easier to control by hospital managers than the outputs [29]. However, considering that structure variables, such as the number of beds, cannot be easily modified, we defined model B as output oriented.

### 2.3 Data analysis

We described patients’ demographics, severity of illness, outcomes, and the organizational characteristics and performance metrics of the ICUs. We used median and interquartile range (IQR, 1^st^ quartile– 3^rd^ quartile) for continuous variables and frequency with respective proportions for categorical variables.

To perform the ICU benchmarking, we executed the three DEA models proposed. Input-oriented models result in efficiency scores that range from 0 to 1, while output-oriented models’ scores range from 1 to infinite. However, in both models, the units are considered efficient when their efficiency score is equal to 1. We obtained the efficiency scores of each ICU and calculated the number of times each efficient unit was considered a reference to others in each model. The selection of reference units depends on the chosen orientation. For example, the reference units to a non-efficient unit in model A (staffing, input-oriented) will be the ones that have similar values of SMR and SRU and that have lower proportions of staffing per 10 beds. On the other hand, in model B (structure, output-oriented), the reference units will be the ones that have similar values of number of beds, physician hours and/or nursing hours and that have lower values of SMR and/or SRU. An explanation of reference units in DEA models is presented in the Supplementary Information.

We compared the results from the DEA models with the traditional ICU benchmarking analysis using the efficiency matrix [3, 23]. In the efficiency matrix, the units were divided into four efficiency groups, depending on their values of SMR and SRU compared to the dataset median: "most efficient" (low SMR and SRU), "overachieving" (low SMR, high SRU), "least efficient" (high SMR and SRU), and "underachieving" (high SMR, low SRU) [3]. We also plotted the DEA results in an SMR versus SRU scatter plot with the efficiency groups for each model.

In DEA models, for each non-efficient unit, there are one or more reference units, which define targets for improvement. The targets of inputs and outputs to a non-efficient unit will be the weighted sum of their reference units’ inputs and outputs. We only presented model A’s targets since its variables are more controllable than the ICU structure (model B) or the bed occupancy rate (model C). These two models are more relevant for comparison between similar units instead of the definition of targets.

Finally, we selected categorical non-discretionary variables to understand the patterns among efficient and non-efficient units after calculating the efficiency scores. We aimed to evaluate the differences in efficiency score according to ICU characteristics that are not in managerial control. We computed the mean score obtained by the units for each category to check if there were differences among units with that specific characteristic. The variables selected for this analysis were the hospital administration (public, private philanthropic, and private for-profit), the type of ICU (not specialized–mixed and medical, and specialized—surgical, neurological, and others), the size of the hospital (small–less than 100 beds, medium–between 100 and 200 beds; =, and large–more than 200 beds) [30], and the proportion of ICU beds. This last variable is the ratio between the number of beds in each ICU and the hospital’s total beds. It was categorized into terciles: low (< = 4.70%), medium (between 4.70% and 9.57%), and high (> = 9.57%) proportions.

We performed the analyses using R software (4.0.1) and the packages *tidyverse* for data management operations and plotting and *deaR* for DEA modeling. We also implemented the DEA models in AIMMS (4.70.3.4) to extract additional results. More information on the DEA model and results are in the supplementary material.

## 3 Results

### 3.1 Characteristics of patients and intensive care units

From 2014 to 2015, 129,680 patients were admitted to 93 ICUs at 55 Brazilian hospitals. The median age was 64 years old, 68% of admissions were medical, and ICU mortality was 12% (**Table 2**).

**Table 2:** Characteristics of the 129,680 patients admitted to the 93 analyzed ICUs.

Characteristic	N = 129,680^1^
**Age**	64 (48, 78)
**Male Gender**	64,018 (49%)
**Admission Type**	
Medical	88,660 (68%)
Scheduled surgery	32,379 (25%)
Emergency surgery	8,641 (6.7%)
**SAPS-3**	44 (34, 54)
**LOS**	
ICU	3 (1, 6)
Hospital	8 (4, 18)
**Mortality**	
ICU	15,606 (12%)
Hospital	23,563 (18%)

^1^Statistics presented: median (IQR); n (%).

Most ICUs are in private for-profit hospitals (61%), are of mixed type (81%), and have less than 30 ICU beds (88%) (**Table 3**). Overall, the median SMR was 1.00, and the median SRU was 1.15. The median physicians per 10 beds ratio was 1.67 (IQR: [1.36–2]), the nurses per 10 beds ratio was 1.71 (IQR: [1.41–2.39]), and the number of nursing technicians per 10 beds was 5 (IQR: [4.55–5.56]).

**Table 3:** Characteristics of the 93 analyzed ICUs.

Characteristics	
**Total ICUs**	93
**Hospital Administration**	
Public	17 (18)
Philanthropic	19 (20)
Private for-profit	57 (61)
**ICU Type**	
Mixed	75 (81)
Surgical	8 (9)
Medical	1 (1)
Neurological	5 (5)
Other	4 (4)
**ICU beds, ICU No (%)**	
<10	37 (40)
11–30	45 (48)
31–50	10 (11)
> 50	1 (1)
**Selected variables**	
SMR	1 [0.79–1.21]
SRU	1.15 [0.95–1.56]
ICU Beds	13 [10–20]
Total Physicians’ hours per week	372 [276–504]
Total Nurses’ hours per week	396 [336–648]
Physician per 10 Beds ratio	1.67 [1.36–2]
Nurse per 10 Beds ratio	1.71 [1.41–2.39]
Nursing Technician per 10 Beds ratio	5 [4.55–5.56]
Physiotherapist per 10 Beds ratio	1 [0.83–1.25]
Bed Occupancy Ratio	0.83 [0.75–0.87]

Statistics presented in n (%) and median [interquartile range].

SMR–Standardized Mortality Ratio; SRU–Standardized Resource Use; ICU–Intensive Care Units.

### 3.2 DEA results

We applied three DEA models: Model A is composed of staffing variables and is input-oriented; model B contains information on the ICU structure and is output-oriented; model C analyses ICU capacity and is input-oriented. All data regarding inputs and outputs used are presented in **S1 Table**. The number of efficient units was 16 (17%) in model A, 8 (9%) in model B, and 10 (11%) in model C (**S2 Table**).

The units considered efficient in model A had a lower median number of physicians per 10 beds when compared to the non-efficient units (**Table 4**). However, the proportion of nurses was higher for the efficient units, and the proportion of nursing technicians and physiotherapists was similar in both groups. The efficient units in model B were much smaller than the non-efficient in terms of the number of ICU beds and physician and nursing hours. The median bed occupancy rate was 92% for the units considered efficient in model C and 83% for the non-efficient. SMR and SRU were lower in the efficient group in all three models.

**Table 4:** Median values (and interquartile ranges, in brackets) of inputs and outputs divided in two groups: efficient and non-efficient units in each model.

		Efficient	Non-efficient
**Model A**			
	Physicians/10 Beds	1.44 [1.18–1.88]	1.7 [1.36–2]
	Nurses/10 Beds	2.02 [1.16–2.48]	1.71 [1.43–2.36]
	Nursing Tec./10 Beds	5 [4.38–5.64]	5 [4.79–5.56]
	Physio/10 Beds	0.98 [0.82–1.16]	1 [0.83–1.25]
	SMR	0.7 [0.57–0.88]	1.07 [0.86–1.2]
	SRU	0.78 [0.61–1.16]	1.2 [0.99–1.61]
**Model B**			
	ICU Beds	8 [6–8.25]	14 [10–20]
	Physicians’ hours	240 [213–276]	384 [276–504]
	Nurses’ hours	168 [168–291]	396 [336–672]
	SMR	0.72 [0.57–1.22]	1.01 [0.81–1.21]
	SRU	0.99 [0.75–1.27]	1.17 [0.95–1.56]
**Model C**			
	Bed Occupancy Rate	0.92 [0.84–0.97]	0.83 [0.76–0.87]
	SMR	0.64 [0.57–0.7]	1.04 [0.84–1.22]
	SRU	1.04 [0.74–1.2]	1.16 [0.95–1.56]

Statistics presented in median [interquartile range].

Nursing Tec./10Beds–number of nursing technicians per 10 beds; Physio/10Beds–number of physiotherapists per 10 beds; SMR–Standardized Mortality Ratio; SRU–Standardized Resource Use.

We evaluated the average efficiency scores for each organizational characteristic of the ICUs and hospitals in the three DEA models (**Table 5**). Units from for-profit hospitals presented the best efficiency scores (values closer to 1) and comprised the most efficient units in all three models (7 to 12 units). Medium-sized hospitals presented the highest efficiency scores in models A and C, whereas model B showed higher efficiency in small-sized hospitals, even though they did not present the lowest average SMR or SRU. Specialized ICUs presented the highest efficient scores and lowest SMR and SRU. The ICUs with a higher proportion of beds in relation to the hospital total also had top results of SMR and SRU.

**Table 5:** Mean efficiency scores in each model, mean SMR and SRU, number of units in each category, and number of units considered efficient in each model. The best results in each category and column are in bold.

	Model A	Model B	Model C	SMR	SRU	n	#Ef A	#Ef B	#Ef C
**Hospital administration**									
Public	0.56	2.25	0.84	1.38	2.10	17	1	0	1
Private, philanthropic	0.71	1.72	0.81	1.09	1.30	19	3	1	2
Private, for-profit	**0.77**	**1.48**	**0.86**	**0.90**	**1.15**	57	12	7	7
**Hospital size**									
Small (< 100 beds)	0.67	**1.64**	0.78	1.04	1.24	9	2	1	1
Medium (≥ 100 and ≤ 200 beds)	**0.75**	1.70	**0.86**	1.03	**1.23**	33	5	2	2
Large (> 200 beds)	0.71	1.66	0.85	**1.02**	1.45	51	9	5	7
**Specialized ICU**									
Yes	**0.77**	**1.44**	**0.87**	**1.01**	**1.07**	17	5	4	3
No	0.71	1.72	0.84	1.03	1.42	76	11	4	7
**Proportion of ICU beds**									
Low (≤ 4.7%)	0.69	**1.63**	**0.85**	1.05	1.53	31	5	6	5
Medium (> 4.7% and < 9.57%)	0.71	1.68	**0.85**	1.02	1.28	31	4	1	2
High (≥ 9.57%)	**0.76**	1.71	0.83	**1.01**	**1.25**	31	7	1	3

**Fig 1:**
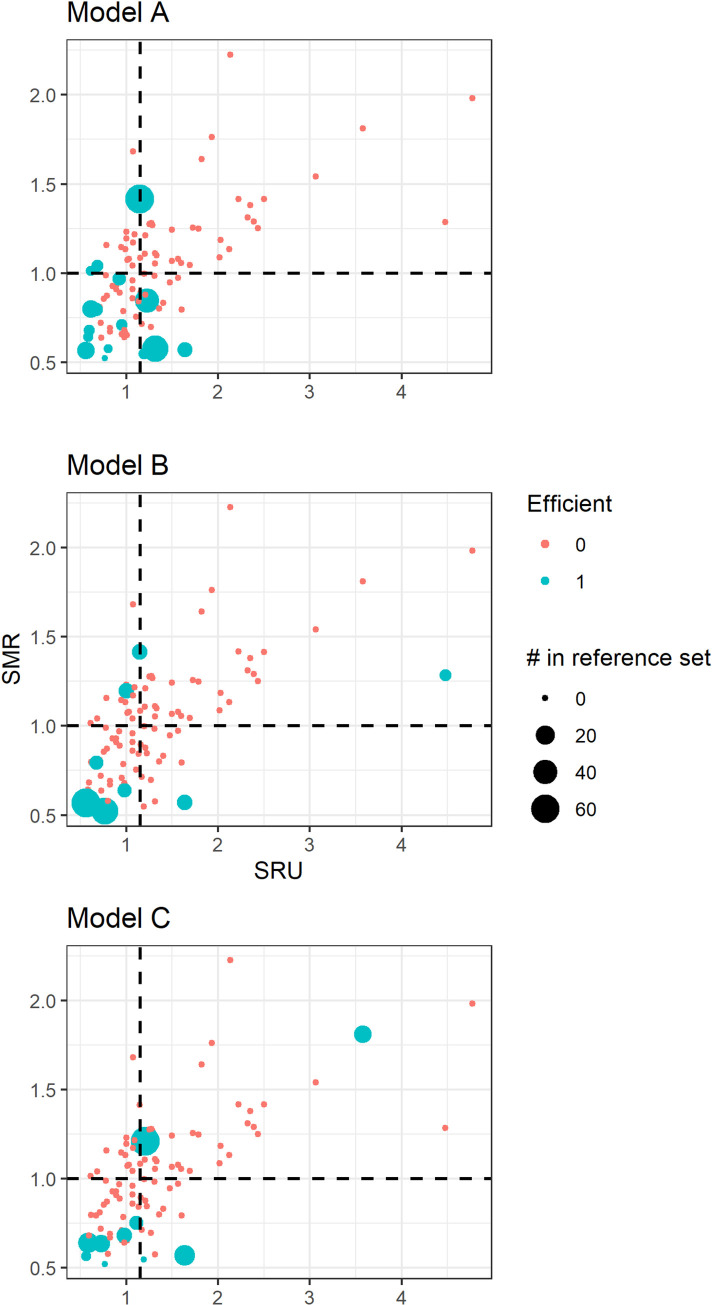
Scatter plot showing all units’ SMR and SRU. The blue color represents the units considered efficient, and the size is proportional to the number of times the unit was considered a reference to others. The dashed lines are the median values of SMR (horizontal) and SRU (vertical).

Comparing the DEA results with the efficiency matrix (**Fig 1**), the SMR values of the DEA efficient units (in blue) are lower than the median, but they are not limited to the "most efficient" group. In models B and C, a few units with high SMR or SRU were considered efficient. The mean efficiency score of units in the "most efficient" group was the highest in all input-oriented models (A and C [0.84 and 0.88, respectively]); and lowest in the output-oriented model (B [1.27]), which is in line with the efficiency matrix analysis (**S3 Table**). The "underachieving" units, however, did not have the worst scores in all DEA models. Three units were considered efficient in all models. Those units had a higher proportion of staffing per 10 beds but presented the number of beds and staffing hours lower than the overall median. Of those units, two presented low occupancy rates (74% and 71%, in comparison to 84% of the dataset median), while one unit had a high occupancy rate (96%). All three units had low SMR and two had low SRU.

We also calculated the targets that each non-efficient unit should aim to achieve in each input and output of Model A (**S4 Table**). The targets are calculated based on the efficient units closest to the non-efficient unit and, therefore, are the shortest path to a unit to be considered efficient by performing changes in its inputs and outputs. Therefore, all non-efficient units in model A, which is input-oriented, should reduce their inputs to be considered efficient. The amount of reduction will depend on the distance of the non-efficient unit to the efficient frontier. An example of the target calculation is presented in **S5 Table**.

## 4 Discussion

We performed benchmarking in a large multicenter dataset of 93 Brazilian ICUs using data envelopment analysis. With three DEA models, we obtained different sets of efficiency units. Efficient ICUs presented lower physicians per bed ratio and nursing workload but higher nurses per beds ratio. DEA results were mostly in line with the efficiency matrix method: generally DEA’s efficiency units were in the "most efficient" quadrant. The use of DEA jointly with the efficiency matrix can provide a deeper understanding of ICU performance and efficiency.

The main strategies for benchmarking in critical care have focused on comparing units with a single metric, such as ranking tables or funnel plots, or combining two outcome measures, as the efficiency matrix. However, the performance of an ICU also depends on the availability of resources. Also, the DEA model compares units individually considering several metrics (outputs) and resources (inputs) without requiring a previous combination such as averaging or performing group comparisons [7]. Our results showed that most DEA-efficient units were also in the "most efficient" group of the efficiency matrix. Thus, managers seeking to purely analyze performance metrics, will achieve accurate results using the efficiency matrix. However, in cases in which the availability of resources (inputs) is relevant to define efficiency, other methods should be considered. DEA allowed units with high values of SMR or SRU to be considered efficient since they presented reasonable levels of inputs for that level of performance.

Therefore, DEA provides hospital managers the information needed to identify the outcomes to be achieved and the levels of resources needed to provide efficient care. For example, in our analysis, units near the "most efficient" quadrant in the efficiency matrix could reduce the inputs to approach the efficient units. Besides, DEA provides a path to hospital managers, as the model can define the levels of inputs and outputs required to be considered efficient (targets).

We considered resources related to staffing, structure, and capacity in this work to analyze different perspectives. The staffing model is helpful to understand whether the level of professionals per 10 beds is compatible with the units’ performance. The structure and capacity models help to compare a unit’s results to other similar units. Besides, other perspectives could be included in the DEA modeling to provide different insights.

We observed that the units considered efficient in all models presented a high staffing-to-bed ratio, even though the number of beds and staffing working load was lower than the medians. This result is in line with previous studies performed not only in Brazil but also in other countries. These works also showed a positive association of staffing-to-bed ratios with high efficient units, especially nurses [21, 31] (in Brazilian and American ICUs, respectively) and intensivists [6] (in Dutch ICUs).

Units from for-profit hospitals had the best efficiency scores in all models. However, in model C, the variation of scores between private and public hospitals was small. Even though public hospitals had poor outcomes, they worked with higher occupancy compared to the private units, similar to previous studies [32]. Also, our studies showed a large variation of scores among models regarding the efficiency per hospital size, but medium and small-sized hospitals presented the best values. Our results agree with Souza et al. [33] as their best efficiency scores from DEA were for medium hospitals (101–200 beds). However, Botega et al. [32] found that large Brazilian hospitals (> 150 beds) had higher efficiency scores, which is probably due to the use of different outputs when compared to ours.

Our study has some strengths and limitations. We evaluated a large multicenter dataset of Brazilian ICUs, which was initially planned for benchmarking purposes [22]. In addition, we used DEA, a frontier analysis method that can identify efficient units using a combination of several outputs (performance metrics) and inputs, without parametric or linear assumptions between the variables. As limitations, we include: first, efficient units were identified considering limited information of inputs and outputs; hence other variables such as patient’s quality of life after being discharged or financial aspects (such as costs and revenues) relevant to ICU managers were not available. However, we included main staffing measurements, risk-adjusted mortality, and use of resources in the model. Second, classic DEA models are entirely numeric, and categorical variables such as characteristics of a unit were not directly considered in the model. However, we could evaluate the efficiency scores obtained from the models for each organizational characteristic and identify possible associations of those aspects with efficiency. Third, we also believe that we can improve our study if we have access to other patient-level data regarding the hospitalization. Fourth, we used deterministic frontier analysis, which might have limitations when compared to stochastic models. Nevertheless, we believe that deterministic DEA is simpler and more intuitive. Since frontier methods have not yet been broadly explored in the ICU efficiency field, we believe that readers could more easily assimilate traditional DEA. Lastly, although some of our results were similar to the ones found in other countries, the Brazilian healthcare system has particularities that might not be extendable globally. On the other hand, the methods and general message of comparison with the efficiency matrix are still useful and applicable to other countries.

## 5 Conclusions

We used severity-adjusted outputs to perform ICU benchmarking in Brazilian hospitals. We had three different perspectives: the staffing patterns, the ICU structure, and ICU capacity. The DEA model had similar results to the efficiency matrix but could provide other insights. Private, for-profit hospitals had better performance in all models and a lower occupancy rate than public hospitals.

## Supporting information

S1 Appendix DEA mathematical model. (DOCX)

S1 Table Inputs and outputs of all units in all three DEA models. (DOCX)

S2 Table Efficiency results of all the DMUs in all models. The values in grey represent the units considered efficient in each model.
(DOCX)

S3 Table Mean efficiency scores and standard deviation of the units present in each quadrant of the efficiency matrix and in each DEA model. (DOCX)

S4 Table Targets (model A). (DOCX)

S5 Table Example of definition of targets for unit 87, considered non-efficient in Model A. (DOCX)

S1 Fig (TIF)
