# Supplementary material for: Using data envelopment analysis to perform benchmarking in intensive care units
Source: PLoS One. 2021 Nov 18;16(11):e0260025. doi: 10.1371/journal.pone.0260025 (PMC8601512; doi:10.1371/journal.pone.0260025)
Supplement: S1 Appendix — (DOCX) [file pone.0260025.s001.docx]

**S1 Appendix – DEA Mathematical model**

Basically, in a DEA model, efficiency is calculated as the ratio of the weighted sum of outputs (outcomes) to the weighted sum of inputs (resources). The weights are defined by maximizing the efficiency of a determined DMU (Eq. 1), with the constraint that each DMU cannot have an efficiency greater than 1 (Eq. 2). Also, the weights have to be non-negative (Eq. 3) (M1) (Charnes, Cooper & Rhodes, 1978).

(M1)

$\max\frac{\sum_{r} \boldsymbol{u}_{\boldsymbol{r}}*\boldsymbol{y}_{\boldsymbol{r}0}}{\sum_{i} \boldsymbol{v}_{\boldsymbol{i}}*\boldsymbol{x}_{\boldsymbol{i}0}}$ (1)

$$s.t.$$

$\frac{\sum_{r} \boldsymbol{u}_{\boldsymbol{r}}*\boldsymbol{y}_{\boldsymbol{rj}}}{\sum_{i} \boldsymbol{v}_{\boldsymbol{i}}*\boldsymbol{x}_{\boldsymbol{ij}}}\leq\mathbf{1} \forall\boldsymbol{j}$ (2)

$\boldsymbol{u}_{\boldsymbol{r}}, \boldsymbol{v}_{\boldsymbol{i}}\geq\mathbf{0} \forall\boldsymbol{r},\boldsymbol{i}$ (3)

Where:

$u_{r}$: weight of each output r

$v_{i}$: weight of each input i

$y_{rj}$: amount of each output r in each DMU j

$x_{ij}$: amount of each input i in each DMU j

The model presented will result in the relative efficiency of one unit (DMU 0). By changing the indices in the objective function, it is possible to analyze the efficiency of each DMU in relation to the rest of the set. Therefore, to obtain the efficiency scores of all units, it is necessary to run the model j times, with j being the number of units in the set.

To linearize the model and to avoid multiple solutions, the denominator of the objective function is set to be equal to one, and the constraints in (2) are also adjusted (the weighted sum of the inputs passes to the right-hand side). This model is known as "multiplier," as there are weights multiplying the input and output values. As it is now a linear programming problem, it has a dual that results in the same value of efficiency (objective function). It is called "envelope," and it is represented by the following equations in model 2 (M2).

$\min h$ (4)

$$s.t.$$

$hx_{i0}\geq\sum_{j} \mu_{j}x_{ij}$ $\forall i$ (5)

$y_{r0}\leq\sum_{j} \mu_{j}y_{rj} \forall r$ (6)

$\mu_{j}\geq0 \forall j$ (7)

Where:

h: efficiency score of DMU 0

$\mu_{j}$: weight of each DMU j

$y_{rj}$: amount of each output r in each DMU j

$x_{ij}$: amount of each input i in each DMU j

In the model above (M2), the maximum efficiency is obtained by reducing the use of inputs and maintaining the outputs constant, defined as input oriented. The objective function (eq. 4) minimizes the efficiency score, which multiplies the inputs of DMU 0. If the efficiency score is equal to one (the maximum value possible in the input-oriented model), it means that there is no need to minimize the inputs, which indicates that the unit is efficient. The output-oriented model, on the other hand, focus on maximizing the use of outputs while maintaining the same level of inputs. Therefore, its efficiency scores range from 1 to infinite, where 1 is the score of an efficient unit.

Also, the models presented are the classic versions, that assume Constant Returns to Scale (CRS), which means that any variation on inputs causes a proportional change in outputs. Another possibility is to consider Variable Returns to Scale (VRS), that assumes that returns to scale can be increasing, constant, or decreasing. Mathematically, the difference is that in the VRS envelope model, there is one more constraint that states that the sum of the weights has to be equal to one (Eq. 8).

$\sum_{j} \mu_{j}=1$ (8)

Therefore. the weights in the multiplier model (M1), define the importance of each input and output to that DMU's efficiency score. That is, if a determined weight of an input is zero, it means that it was not considered to calculate the score of that unit, probably because it was too high when compared to the other DMUs' values. On the other hand, the weights in the envelope model (M2) represent the importance of each DMU as a reference to the unit being analyzed. Thus, if a DMU is considered efficient, it will have a weight of 1 to itself and zero to the other units.

These weights can be useful to define the reference sets, which can be relevant to differentiate among efficient DMUs. Some units can be efficient and a reference to others, while others can be efficient but not a reference to any DMU. The reference sets will depend on the model orientation. A graphic representation of theoretical units in a model that only has one input and one output is shown in Figure S1.

Figure S1 – Representation of the reference set

In the example above, DMU E is not efficient, and, in an input-oriented model, its references would be DMUs B and C, as the model maintains outputs constant and reduces the inputs. In an output-oriented model, however, the outputs would be maximized while the inputs would stay constant. In that case, the reference set of DMU E would be composed of DMUs C and D.
