# Supplementary material for: Using data envelopment analysis to perform benchmarking in intensive care units
Source: PLoS One. 2021 Nov 18;16(11):e0260025. doi: 10.1371/journal.pone.0260025 (PMC8601512; doi:10.1371/journal.pone.0260025)
Supplement: S1 Table — (DOCX) [file pone.0260025.s002.docx]

S1 Table – Inputs and outputs of all units in all three DEA models

| **ICU** | **A** | | | | **B** | | | **C** | **Outputs** | |
| --- | --- | --- | --- | --- | --- | --- | --- | --- | --- | --- |
|  | **MD_ Bed10** | **Nur_ Bed10** | **NurTec_ Bed10** | **Physio_ Bed10** | **ICU_ Bed** | **MD_ hours** | **Nur_ hours** | **BOR** | **SMR** | **SRU** |
| 1 | 1.64 | 1.55 | 4.79 | 1.25 | 24 | 660 | 624 | 0.94 | 1.26 | 1.73 |
| 2 | 1.76 | 1.81 | 5.00 | 1.54 | 13 | 384 | 396 | 0.91 | 1.27 | 1.28 |
| 3 | 1.64 | 1.36 | 5.00 | 1.00 | 10 | 276 | 228 | 0.87 | 1.23 | 1.00 |
| 4 | 1.36 | 2.36 | 5.00 | 0.00 | 10 | 228 | 396 | 0.75 | 0.71 | 0.95 |
| 5 | 1.76 | 2.39 | 4.71 | 1.55 | 17 | 504 | 684 | 0.87 | 1.42 | 2.50 |
| 6 | 2.05 | 1.70 | 6.88 | 1.25 | 8 | 276 | 228 | 0.91 | 1.64 | 1.82 |
| 7 | 2.00 | 2.71 | 6.50 | 1.36 | 10 | 336 | 456 | 0.97 | 1.09 | 2.01 |
| 8 | 1.67 | 2.80 | 5.00 | 0.83 | 12 | 336 | 564 | 0.74 | 0.89 | 0.93 |
| 9 | 2.54 | 2.62 | 4.44 | 1.11 | 9 | 384 | 396 | 0.83 | 0.79 | 0.97 |
| 10 | 2.71 | 2.00 | 4.00 | 2.00 | 5 | 228 | 168 | 0.75 | 1.20 | 1.00 |
| 11 | 2.86 | 3.37 | 5.00 | 1.43 | 7 | 336 | 396 | 0.74 | 0.93 | 0.89 |
| 12 | 2.36 | 2.36 | 4.00 | 1.00 | 10 | 396 | 396 | 0.68 | 0.99 | 0.78 |
| 13 | 2.50 | 2.95 | 5.00 | 1.25 | 8 | 336 | 396 | 0.79 | 0.87 | 0.79 |
| 14 | 1.82 | 2.47 | 4.55 | 0.91 | 11 | 336 | 456 | 0.70 | 1.01 | 0.61 |
| 15 | 1.18 | 0.68 | 4.50 | 0.68 | 20 | 420 | 228 | 0.87 | 0.97 | 0.92 |
| 16 | 1.23 | 3.07 | 4.09 | 0.69 | 33 | 684 | 1704 | 0.96 | 0.70 | 1.27 |
| 17 | 1.86 | 1.54 | 4.00 | 0.82 | 20 | 624 | 516 | 0.75 | 1.16 | 0.78 |
| 18 | 1.24 | 3.18 | 14.21 | 1.13 | 38 | 816 | 2052 | 0.84 | 0.64 | 0.58 |
| 19 | 1.45 | 2.43 | 5.95 | 1.60 | 37 | 924 | 1536 | 0.83 | 0.68 | 0.60 |
| 20 | 1.23 | 2.14 | 5.45 | 1.23 | 11 | 228 | 396 | 0.87 | 2.23 | 2.13 |
| 21 | 1.43 | 2.67 | 5.33 | 1.45 | 30 | 744 | 1368 | 0.79 | 0.85 | 0.76 |
| 22 | 1.36 | 1.18 | 5.00 | 1.00 | 20 | 456 | 396 | 1.04 | 1.21 | 1.21 |
| 23 | 2.29 | 2.36 | 4.00 | 1.00 | 10 | 384 | 396 | 0.75 | 0.80 | 0.62 |
| 24 | 2.05 | 2.50 | 5.00 | 1.25 | 8 | 276 | 336 | 0.74 | 0.57 | 0.56 |
| 25 | 2.05 | 2.50 | 5.00 | 1.25 | 8 | 276 | 336 | 0.89 | 0.64 | 0.73 |
| 26 | 2.05 | 2.50 | 5.00 | 1.25 | 8 | 276 | 336 | 0.90 | 0.72 | 0.72 |
| 27 | 2.00 | 2.00 | 4.00 | 1.00 | 10 | 336 | 336 | 0.89 | 0.67 | 0.82 |
| 28 | 2.05 | 2.50 | 5.00 | 1.25 | 8 | 276 | 336 | 0.90 | 0.69 | 0.82 |
| 29 | 1.96 | 2.50 | 5.00 | 0.83 | 12 | 396 | 504 | 0.69 | 0.81 | 0.71 |
| 30 | 1.28 | 1.58 | 5.59 | 1.28 | 34 | 756 | 924 | 0.83 | 0.89 | 1.16 |
| 31 | 2.00 | 2.36 | 5.00 | 0.50 | 20 | 672 | 792 | 0.78 | 0.66 | 0.95 |
| 34 | 2.10 | 1.69 | 5.00 | 1.36 | 30 | 1056 | 852 | 0.83 | 0.93 | 0.85 |
| 35 | 1.18 | 1.71 | 5.25 | 1.18 | 20 | 396 | 576 | 0.92 | 1.06 | 1.60 |
| 36 | 1.51 | 2.22 | 3.89 | 1.11 | 18 | 456 | 672 | 0.84 | 0.91 | 1.07 |
| 37 | 1.65 | 1.47 | 5.00 | 0.40 | 16 | 444 | 396 | 0.76 | 1.04 | 1.07 |
| 38 | 1.47 | 1.25 | 5.00 | 0.85 | 16 | 396 | 336 | 0.73 | 1.10 | 1.33 |
| 39 | 1.43 | 3.83 | 1.43 | 0.97 | 14 | 336 | 900 | 0.54 | 0.85 | 1.23 |
| 40 | 1.70 | 1.70 | 5.62 | 1.25 | 8 | 228 | 228 | 0.85 | 1.00 | 1.20 |
| 41 | 1.70 | 2.14 | 5.62 | 1.25 | 8 | 228 | 288 | 0.86 | 1.28 | 1.25 |
| 42 | 1.70 | 2.14 | 5.62 | 1.25 | 8 | 228 | 288 | 0.84 | 1.28 | 1.27 |
| 43 | 1.36 | 1.71 | 5.50 | 1.00 | 10 | 228 | 288 | 0.89 | 1.54 | 3.06 |
| 44 | 1.00 | 1.18 | 5.00 | 1.00 | 20 | 360 | 396 | 0.78 | 1.08 | 1.56 |
| 45 | 1.67 | 1.67 | 3.33 | 1.67 | 6 | 168 | 168 | 0.73 | 1.29 | 4.48 |
| 46 | 1.05 | 1.05 | 5.26 | 1.05 | 19 | 336 | 336 | 0.78 | 1.11 | 1.31 |
| 47 | 0.97 | 1.43 | 5.00 | 0.71 | 14 | 228 | 336 | 0.76 | 1.09 | 1.15 |
| 48 | 1.43 | 1.43 | 2.86 | 0.71 | 28 | 672 | 672 | 0.75 | 1.76 | 1.94 |
| 49 | 2.14 | 1.82 | 4.55 | 0.58 | 11 | 396 | 336 | 1.04 | 1.81 | 3.58 |
| 50 | 2.00 | 2.71 | 5.00 | 0.64 | 10 | 336 | 456 | 1.01 | 1.98 | 4.77 |
| 51 | 0.86 | 1.35 | 3.71 | 0.86 | 35 | 504 | 816 | 0.88 | 0.58 | 1.31 |
| 52 | 1.89 | 1.68 | 3.57 | 0.71 | 14 | 444 | 396 | 0.80 | 1.04 | 1.70 |
| 53 | 2.03 | 1.54 | 3.85 | 0.77 | 13 | 444 | 336 | 0.83 | 1.15 | 0.95 |
| 54 | 1.89 | 1.43 | 3.57 | 0.71 | 14 | 444 | 336 | 0.79 | 1.25 | 1.79 |
| 55 | 1.09 | 1.25 | 5.50 | 0.91 | 40 | 732 | 840 | 0.78 | 1.11 | 1.20 |
| 56 | 2.05 | 2.50 | 4.06 | 0.85 | 16 | 552 | 672 | 0.98 | 0.88 | 1.21 |
| 57 | 1.83 | 2.22 | 4.44 | 1.11 | 9 | 276 | 336 | 0.98 | 0.75 | 1.11 |
| 58 | 2.74 | 3.33 | 5.00 | 1.67 | 6 | 276 | 336 | 0.71 | 0.52 | 0.77 |
| 59 | 1.64 | 2.71 | 5.00 | 1.36 | 10 | 276 | 456 | 0.65 | 1.07 | 1.02 |
| 60 | 1.64 | 2.00 | 4.00 | 1.00 | 10 | 276 | 336 | 0.52 | 0.84 | 1.14 |
| 61 | 1.00 | 1.12 | 5.33 | 0.88 | 30 | 504 | 564 | 0.79 | 0.71 | 1.17 |
| 62 | 1.64 | 1.36 | 5.00 | 1.00 | 10 | 276 | 228 | 0.87 | 1.13 | 2.12 |
| 63 | 1.83 | 1.51 | 5.56 | 1.11 | 9 | 276 | 228 | 0.86 | 0.98 | 1.31 |
| 64 | 1.88 | 1.47 | 5.62 | 1.25 | 16 | 504 | 396 | 0.75 | 0.86 | 1.07 |
| 65 | 2.26 | 2.74 | 7.08 | 1.67 | 12 | 480 | 552 | 0.84 | 1.18 | 2.03 |
| 66 | 1.24 | 1.43 | 5.26 | 0.53 | 19 | 396 | 456 | 0.78 | 1.68 | 1.08 |
| 67 | 1.51 | 1.11 | 7.22 | 0.71 | 9 | 228 | 168 | 0.85 | 1.25 | 2.43 |
| 68 | 1.51 | 1.11 | 7.22 | 0.71 | 9 | 228 | 168 | 0.85 | 1.42 | 2.22 |
| 69 | 1.23 | 1.56 | 5.91 | 0.58 | 11 | 228 | 288 | 0.85 | 1.29 | 2.39 |
| 70 | 2.46 | 1.11 | 5.56 | 1.11 | 9 | 372 | 168 | 0.87 | 0.66 | 0.99 |
| 71 | 2.46 | 1.11 | 5.56 | 1.11 | 9 | 372 | 168 | 0.85 | 0.79 | 0.67 |
| 72 | 1.85 | 0.83 | 5.00 | 0.83 | 12 | 372 | 168 | 0.83 | 0.96 | 1.07 |
| 74 | 2.00 | 3.14 | 6.00 | 1.00 | 10 | 360 | 528 | 0.84 | 1.07 | 1.50 |
| 75 | 1.43 | 1.43 | 6.43 | 0.97 | 14 | 360 | 336 | 0.88 | 1.17 | 1.07 |
| 76 | 1.82 | 1.82 | 7.73 | 0.91 | 11 | 360 | 336 | 0.88 | 1.24 | 1.50 |
| 77 | 1.21 | 1.45 | 5.33 | 1.00 | 30 | 612 | 732 | 0.82 | 0.65 | 1.00 |
| 78 | 1.63 | 1.14 | 4.00 | 0.94 | 35 | 984 | 672 | 0.82 | 1.13 | 0.99 |
| 79 | 1.03 | 1.25 | 5.00 | 0.00 | 16 | 276 | 336 | 0.85 | 1.05 | 1.31 |
| 80 | 0.62 | 0.62 | 2.81 | 0.00 | 16 | 168 | 168 | 0.62 | 1.41 | 1.15 |
| 81 | 1.36 | 1.36 | 5.00 | 1.00 | 10 | 228 | 228 | 0.95 | 0.91 | 0.89 |
| 82 | 1.55 | 1.68 | 5.88 | 0.78 | 34 | 888 | 984 | 0.79 | 0.58 | 0.80 |
| 83 | 0.99 | 1.41 | 4.61 | 0.84 | 64 | 1068 | 1512 | 0.86 | 0.55 | 1.19 |
| 84 | 1.70 | 0.80 | 5.00 | 1.25 | 8 | 252 | 108 | 0.96 | 0.57 | 1.64 |
| 85 | 1.07 | 0.88 | 4.55 | 1.07 | 22 | 420 | 324 | 0.85 | 0.80 | 1.36 |
| 86 | 1.33 | 1.81 | 5.33 | 1.57 | 15 | 360 | 456 | 0.95 | 0.68 | 0.98 |
| 87 | 0.78 | 1.60 | 5.88 | 0.80 | 34 | 444 | 912 | 0.65 | 1.22 | 1.09 |
| 88 | 2.69 | 2.20 | 6.86 | 1.04 | 35 | 1584 | 1296 | 0.90 | 1.31 | 2.33 |
| 89 | 2.07 | 1.97 | 6.90 | 1.03 | 29 | 1008 | 960 | 0.54 | 1.38 | 2.35 |
| 90 | 1.31 | 1.71 | 5.40 | 1.06 | 25 | 552 | 720 | 0.86 | 0.83 | 1.40 |
| 91 | 1.46 | 1.71 | 5.40 | 1.06 | 25 | 636 | 720 | 0.85 | 0.95 | 1.48 |
| 92 | 1.64 | 2.00 | 5.50 | 1.00 | 10 | 276 | 336 | 0.79 | 0.79 | 1.61 |
| 93 | 1.70 | 2.05 | 5.62 | 1.25 | 8 | 228 | 276 | 0.66 | 0.64 | 0.98 |
| 95 | 1.18 | 1.18 | 6.00 | 1.00 | 20 | 396 | 396 | 0.78 | 1.04 | 0.68 |
| 96 | 2.26 | 3.33 | 5.00 | 1.07 | 6 | 252 | 336 | 0.67 | 1.08 | 1.03 |
| 97 | 1.88 | 1.25 | 5.62 | 1.47 | 16 | 504 | 336 | 0.94 | 0.97 | 1.56 |
