# Supplementary material for: Using data envelopment analysis to perform benchmarking in intensive care units
Source: PLoS One. 2021 Nov 18;16(11):e0260025. doi: 10.1371/journal.pone.0260025 (PMC8601512; doi:10.1371/journal.pone.0260025)
Supplement: S2 Table — The values in grey represent the units considered efficient in each model. (DOCX) [file pone.0260025.s003.docx]

S2 Table - Efficiency results of all the DMUs in all models. The values in grey represent the units considered efficient in each model.

| **DMU** | **A** | | **B** | | **C** | |
| --- | --- | --- | --- | --- | --- | --- |
|  | **# ref** | **Score** | **# ref** | **Score** | **#ref** | **Score** |
| 1 | - | 0.576 | - | 2.376 | - | 0.911 |
| 2 | - | 0.539 | - | 2.252 | - | 0.879 |
| 3 | - | 0.628 | - | 1.402 | - | 0.880 |
| 4 | 4 | 1.000 | - | 1.059 | - | 0.788 |
| 5 | - | 0.536 | - | 2.712 | - | 0.842 |
| 6 | - | 0.404 | - | 2.436 | - | 0.881 |
| 7 | - | 0.425 | - | 2.082 | - | 0.945 |
| 8 | - | 0.652 | - | 1.591 | - | 0.764 |
| 9 | - | 0.719 | - | 1.455 | - | 0.857 |
| 10 | - | 0.704 | 10 | 1.000 | - | 0.753 |
| 11 | - | 0.597 | - | 1.375 | - | 0.773 |
| 12 | - | 0.829 | - | 1.389 | - | 0.737 |
| 13 | - | 0.676 | - | 1.408 | - | 0.855 |
| 14 | 2 | 1.000 | - | 1.087 | - | 0.818 |
| 15 | 8 | 1.000 | - | 1.428 | - | 0.902 |
| 16 | - | 0.847 | - | 1.335 | - | 0.981 |
| 17 | - | 0.929 | - | 1.395 | - | 0.816 |
| 18 | 2 | 1.000 | - | 1.043 | 27 | 1.000 |
| 19 | 3 | 1.000 | - | 1.065 | - | 0.986 |
| 20 | - | 0.512 | - | 2.868 | - | 0.833 |
| 21 | - | 0.814 | - | 1.355 | - | 0.865 |
| 22 | - | 0.588 | - | 2.142 | 75 | 1.000 |
| 23 | 19 | 1.000 | - | 1.100 | - | 0.880 |
| 24 | 18 | 1.000 | 63 | 1.000 | 2 | 1.000 |
| 25 | - | 0.835 | - | 1.162 | 19 | 1.000 |
| 26 | - | 0.796 | - | 1.276 | - | 0.997 |
| 27 | - | 0.963 | - | 1.241 | - | 0.966 |
| 28 | - | 0.759 | - | 1.272 | - | 0.977 |
| 29 | - | 0.826 | - | 1.267 | - | 0.771 |
| 30 | - | 0.591 | - | 1.672 | - | 0.834 |
| 31 | - | 0.888 | - | 1.256 | - | 0.836 |
| 34 | - | 0.726 | - | 1.520 | - | 0.880 |
| 35 | - | 0.574 | - | 2.022 | - | 0.893 |
| 36 | - | 0.742 | - | 1.671 | - | 0.850 |
| 37 | - | 0.626 | - | 1.856 | - | 0.759 |
| 38 | - | 0.602 | - | 2.029 | - | 0.712 |
| 39 | 45 | 1.000 | - | 1.616 | - | 0.544 |
| 40 | - | 0.537 | - | 1.271 | - | 0.832 |
| 41 | - | 0.474 | - | 1.458 | - | 0.833 |
| 42 | - | 0.474 | - | 1.479 | - | 0.808 |
| 43 | - | 0.495 | - | 2.405 | - | 0.861 |
| 44 | - | 0.671 | - | 2.062 | - | 0.759 |
| 45 | - | 0.760 | - | 1.000 | - | 0.699 |
| 46 | - | 0.686 | - | 2.041 | - | 0.756 |
| 47 | - | 0.699 | - | 1.482 | - | 0.741 |
| 48 | - | 0.885 | - | 3.188 | - | 0.725 |
| 49 | - | 0.577 | - | 3.470 | 18 | 1.000 |
| 50 | - | 0.499 | - | 3.797 | - | 0.968 |
| 51 | 57 | 1.000 | - | 1.103 | - | 0.940 |
| 52 | - | 0.764 | - | 2.001 | - | 0.781 |
| 53 | - | 0.791 | - | 1.691 | - | 0.849 |
| 54 | - | 0.744 | - | 2.379 | - | 0.757 |
| 55 | - | 0.611 | - | 1.999 | - | 0.761 |
| 56 | - | 0.671 | - | 1.662 | - | 0.981 |
| 57 | - | 0.721 | - | 1.445 | 9 | 1.000 |
| 58 | 0 | 1.000 | 51 | 1.000 | 0 | 1.000 |
| 59 | - | 0.577 | - | 1.812 | - | 0.651 |
| 60 | - | 0.751 | - | 1.589 | - | 0.527 |
| 61 | - | 0.935 | - | 1.367 | - | 0.815 |
| 62 | - | 0.586 | - | 2.080 | - | 0.843 |
| 63 | - | 0.555 | - | 1.681 | - | 0.845 |
| 64 | - | 0.645 | - | 1.596 | - | 0.760 |
| 65 | - | 0.384 | - | 2.270 | - | 0.810 |
| 66 | - | 0.566 | - | 1.919 | - | 0.769 |
| 67 | - | 0.573 | - | 1.836 | - | 0.820 |
| 68 | - | 0.559 | - | 1.896 | - | 0.815 |
| 69 | - | 0.517 | - | 2.014 | - | 0.821 |
| 70 | - | 0.923 | - | 1.068 | - | 0.925 |
| 71 | 7 | 1.000 | 7 | 1.000 | - | 0.961 |
| 72 | - | 0.819 | - | 1.375 | - | 0.829 |
| 74 | - | 0.440 | - | 2.029 | - | 0.820 |
| 75 | - | 0.517 | - | 1.919 | - | 0.871 |
| 76 | - | 0.376 | - | 2.292 | - | 0.847 |
| 77 | - | 0.875 | - | 1.251 | - | 0.870 |
| 78 | - | 0.795 | - | 1.770 | - | 0.831 |
| 79 | - | 0.976 | - | 1.957 | - | 0.825 |
| 80 | 74 | 1.000 | 11 | 1.000 | - | 0.603 |
| 81 | - | 0.797 | - | 1.094 | - | 0.992 |
| 82 | 1 | 1.000 | - | 1.096 | - | 0.941 |
| 83 | 3 | 1.000 | - | 1.050 | 0 | 1.000 |
| 84 | 11 | 1.000 | 11 | 1.000 | 30 | 1.000 |
| 85 | - | 0.943 | - | 1.525 | - | 0.848 |
| 86 | - | 0.768 | - | 1.299 | 13 | 1.000 |
| 87 | - | 0.864 | - | 1.945 | - | 0.643 |
| 88 | - | 0.397 | - | 2.513 | - | 0.865 |
| 89 | - | 0.397 | - | 2.646 | - | 0.524 |
| 90 | - | 0.595 | - | 1.592 | - | 0.859 |
| 91 | - | 0.565 | - | 1.814 | - | 0.838 |
| 92 | - | 0.581 | - | 1.523 | - | 0.787 |
| 93 | - | 0.703 | 7 | 1.000 | - | 0.703 |
| 95 | 5 | 1.000 | - | 1.216 | - | 0.882 |
| 96 | - | 0.527 | - | 1.305 | - | 0.667 |
| 97 | - | 0.593 | - | 1.862 | - | 0.928 |
