# Supplementary material for: Using data envelopment analysis to perform benchmarking in intensive care units
Source: PLoS One. 2021 Nov 18;16(11):e0260025. doi: 10.1371/journal.pone.0260025 (PMC8601512; doi:10.1371/journal.pone.0260025)
Supplement: S3 Table — (DOCX) [file pone.0260025.s004.docx]

S3 Table – Mean efficiency scores and standard deviation of the units present in each quadrant of the efficiency matrix and in each DEA model.

| **Efficiency Matrix** | **DEA score** | | |
| --- | --- | --- | --- |
|  | **Model A** | **Model B** | **Model C** |
| **Most efficient** (SMR < 1 and SRU < 1) | 0.84 (0.13) | 1.27 (0.2) | 0.88 (0.11) |
| **Overachieving** (SMR < 1 and SRU > 1) | 0.76 (0.2) | 1.47 (0.27) | 0.87 (0.12) |
| **Least efficient** (SMR > 1 and SRU > 1) | 0.57 (0.14) | 2.23 (0.56) | 0.82 (0.09) |
| **Underachieving** (SMR > 1 and SRU < 1) | 0.75 (0.18) | 1.52 (0.36) | 0.77 (0.1) |
