# Supplementary material for: Using data envelopment analysis to perform benchmarking in intensive care units
Source: PLoS One. 2021 Nov 18;16(11):e0260025. doi: 10.1371/journal.pone.0260025 (PMC8601512; doi:10.1371/journal.pone.0260025)
Supplement: S4 Table — (DOCX) [file pone.0260025.s005.docx]

S4 Table – Targets (model A)

| **DMU** | **MD_Bed10** | **Nur_Bed10** | **NurTec_Bed10** | **Physio_Bed10** | **SMR** | **SRU** |
| --- | --- | --- | --- | --- | --- | --- |
| 1 | 0.69 | 0.89 | 2.76 | 0.12 | 1.26 | 1.16 |
| 2 | 0.71 | 0.98 | 2.70 | 0.13 | 1.27 | 1.16 |
| 3 | 0.93 | 0.85 | 3.14 | 0.19 | 1.23 | 1.00 |
| 4 | 1.36 | 2.36 | 5.00 | 0.00 | 0.71 | 0.95 |
| 5 | 0.79 | 1.28 | 2.53 | 0.20 | 1.24 | 1.16 |
| 6 | 0.64 | 0.69 | 2.78 | 0.02 | 1.40 | 1.15 |
| 7 | 0.76 | 1.15 | 2.76 | 0.25 | 1.09 | 1.18 |
| 8 | 1.09 | 1.54 | 3.26 | 0.51 | 0.89 | 0.93 |
| 9 | 1.29 | 1.88 | 3.19 | 0.72 | 0.79 | 0.97 |
| 10 | 1.04 | 1.41 | 2.82 | 0.32 | 1.14 | 1.00 |
| 11 | 1.42 | 2.01 | 2.99 | 0.63 | 0.93 | 0.89 |
| 12 | 1.65 | 1.96 | 3.32 | 0.67 | 0.94 | 0.78 |
| 13 | 1.65 | 2.00 | 3.38 | 0.73 | 0.87 | 0.79 |
| 14 | 1.82 | 2.47 | 4.55 | 0.91 | 1.01 | 0.61 |
| 15 | 1.18 | 0.68 | 4.50 | 0.68 | 0.97 | 0.92 |
| 16 | 1.04 | 1.93 | 3.46 | 0.58 | 0.70 | 1.18 |
| 17 | 1.60 | 1.43 | 3.72 | 0.59 | 0.98 | 0.78 |
| 18 | 1.24 | 3.18 | 14.21 | 1.13 | 0.64 | 0.58 |
| 19 | 1.45 | 2.43 | 5.95 | 1.60 | 0.68 | 0.60 |
| 20 | 0.63 | 0.66 | 2.79 | 0.01 | 1.40 | 1.15 |
| 21 | 1.16 | 1.60 | 4.34 | 0.80 | 0.85 | 0.76 |
| 22 | 0.66 | 0.69 | 2.94 | 0.11 | 1.21 | 1.17 |
| 23 | 2.29 | 2.36 | 4.00 | 1.00 | 0.80 | 0.62 |
| 24 | 2.05 | 2.50 | 5.00 | 1.25 | 0.57 | 0.56 |
| 25 | 1.71 | 2.09 | 4.17 | 1.02 | 0.64 | 0.73 |
| 26 | 1.63 | 1.99 | 3.98 | 0.86 | 0.72 | 0.72 |
| 27 | 1.61 | 1.93 | 3.85 | 0.94 | 0.67 | 0.82 |
| 28 | 1.56 | 1.90 | 3.80 | 0.89 | 0.69 | 0.82 |
| 29 | 1.62 | 1.94 | 4.13 | 0.69 | 0.81 | 0.71 |
| 30 | 0.76 | 0.93 | 3.30 | 0.38 | 0.89 | 1.16 |
| 31 | 1.29 | 2.10 | 4.44 | 0.44 | 0.66 | 0.95 |
| 34 | 1.15 | 1.23 | 3.63 | 0.45 | 0.93 | 0.85 |
| 35 | 0.68 | 0.80 | 3.02 | 0.20 | 1.06 | 1.18 |
| 36 | 1.05 | 1.65 | 2.89 | 0.51 | 0.91 | 1.07 |
| 37 | 0.80 | 0.92 | 3.13 | 0.25 | 1.04 | 1.07 |
| 38 | 0.68 | 0.75 | 3.01 | 0.18 | 1.10 | 1.18 |
| 39 | 1.43 | 3.83 | 1.43 | 0.97 | 0.85 | 1.23 |
| 40 | 0.71 | 0.91 | 3.02 | 0.26 | 1.00 | 1.19 |
| 41 | 0.72 | 1.01 | 2.66 | 0.13 | 1.28 | 1.16 |
| 42 | 0.72 | 1.01 | 2.66 | 0.13 | 1.28 | 1.16 |
| 43 | 0.67 | 0.83 | 2.72 | 0.06 | 1.36 | 1.15 |
| 44 | 0.67 | 0.78 | 3.00 | 0.18 | 1.08 | 1.18 |
| 45 | 0.78 | 1.27 | 2.53 | 0.20 | 1.25 | 1.16 |
| 46 | 0.72 | 0.72 | 3.06 | 0.19 | 1.11 | 1.19 |
| 47 | 0.68 | 0.81 | 3.20 | 0.19 | 1.09 | 1.15 |
| 48 | 0.78 | 1.27 | 2.53 | 0.20 | 1.25 | 1.16 |
| 49 | 0.73 | 1.05 | 2.63 | 0.13 | 1.30 | 1.16 |
| 50 | 0.80 | 1.35 | 2.50 | 0.22 | 1.23 | 1.16 |
| 51 | 0.86 | 1.35 | 3.71 | 0.86 | 0.58 | 1.31 |
| 52 | 0.80 | 1.28 | 2.73 | 0.31 | 1.04 | 1.19 |
| 53 | 1.08 | 1.22 | 3.04 | 0.30 | 1.15 | 0.95 |
| 54 | 0.73 | 1.06 | 2.66 | 0.15 | 1.25 | 1.16 |
| 55 | 0.67 | 0.76 | 2.98 | 0.16 | 1.11 | 1.18 |
| 56 | 0.91 | 1.68 | 2.72 | 0.51 | 0.88 | 1.21 |
| 57 | 1.04 | 1.60 | 3.20 | 0.66 | 0.75 | 1.11 |
| 58 | 2.74 | 3.33 | 5.00 | 1.67 | 0.52 | 0.77 |
| 59 | 0.95 | 1.40 | 2.89 | 0.33 | 1.07 | 1.02 |
| 60 | 0.95 | 1.50 | 3.00 | 0.53 | 0.84 | 1.14 |
| 61 | 0.94 | 1.05 | 3.82 | 0.70 | 0.71 | 1.17 |
| 62 | 0.67 | 0.80 | 2.93 | 0.16 | 1.13 | 1.17 |
| 63 | 0.69 | 0.84 | 3.08 | 0.26 | 0.98 | 1.19 |
| 64 | 1.00 | 0.95 | 3.62 | 0.50 | 0.86 | 1.07 |
| 65 | 0.73 | 1.05 | 2.72 | 0.18 | 1.18 | 1.17 |
| 66 | 0.70 | 0.76 | 2.97 | 0.09 | 1.35 | 1.08 |
| 67 | 0.72 | 0.64 | 3.00 | 0.11 | 1.25 | 1.18 |
| 68 | 0.62 | 0.62 | 2.81 | 0.00 | 1.41 | 1.15 |
| 69 | 0.64 | 0.67 | 2.87 | 0.06 | 1.29 | 1.16 |
| 70 | 1.72 | 1.02 | 5.13 | 1.02 | 0.66 | 0.99 |
| 71 | 2.46 | 1.11 | 5.56 | 1.11 | 0.79 | 0.67 |
| 72 | 1.08 | 0.68 | 4.01 | 0.55 | 0.96 | 1.07 |
| 74 | 0.82 | 1.38 | 2.64 | 0.31 | 1.07 | 1.18 |
| 75 | 0.74 | 0.74 | 3.33 | 0.22 | 1.17 | 1.07 |
| 76 | 0.65 | 0.68 | 2.91 | 0.09 | 1.24 | 1.16 |
| 77 | 1.06 | 1.27 | 4.66 | 0.85 | 0.65 | 1.00 |
| 78 | 0.86 | 0.91 | 3.18 | 0.21 | 1.13 | 0.99 |
| 79 | 0.88 | 1.22 | 3.57 | 0.00 | 1.05 | 1.07 |
| 80 | 0.62 | 0.62 | 2.81 | 0.00 | 1.41 | 1.15 |
| 81 | 1.08 | 1.08 | 3.99 | 0.56 | 0.91 | 0.89 |
| 82 | 1.55 | 1.68 | 5.88 | 0.78 | 0.58 | 0.80 |
| 83 | 0.99 | 1.41 | 4.61 | 0.84 | 0.55 | 1.19 |
| 84 | 1.70 | 0.80 | 5.00 | 1.25 | 0.57 | 1.64 |
| 85 | 1.01 | 0.83 | 3.69 | 0.57 | 0.80 | 1.30 |
| 86 | 1.02 | 1.39 | 4.09 | 0.82 | 0.68 | 0.98 |
| 87 | 0.67 | 0.83 | 3.59 | 0.12 | 1.22 | 1.09 |
| 88 | 0.69 | 0.87 | 2.72 | 0.09 | 1.31 | 1.16 |
| 89 | 0.66 | 0.78 | 2.74 | 0.05 | 1.37 | 1.15 |
| 90 | 0.75 | 1.02 | 3.21 | 0.42 | 0.83 | 1.22 |
| 91 | 0.73 | 0.97 | 3.05 | 0.31 | 0.95 | 1.20 |
| 92 | 0.79 | 1.16 | 3.20 | 0.49 | 0.79 | 1.23 |
| 93 | 1.16 | 1.44 | 3.95 | 0.83 | 0.64 | 0.98 |
| 95 | 1.18 | 1.18 | 6.00 | 1.00 | 1.04 | 0.68 |
| 96 | 1.10 | 1.75 | 2.63 | 0.42 | 1.08 | 1.03 |
| 97 | 0.85 | 0.74 | 3.33 | 0.34 | 0.97 | 1.24 |
