# Supplementary material for: Using data envelopment analysis to perform benchmarking in intensive care units
Source: PLoS One. 2021 Nov 18;16(11):e0260025. doi: 10.1371/journal.pone.0260025 (PMC8601512; doi:10.1371/journal.pone.0260025)
Supplement: S5 Table — (DOCX) [file pone.0260025.s006.docx]

S5 Table - Example of definition of targets for unit 87, considered non-efficient in Model A. The first line presents its actual values. For this unit, the reference ICUs in the model were 18, 51, and 80, representing respectively 6.3%, 6%, 87.6% of the reference (last column), which sum 100%. The targets are the weigthed sum of these percentages multiplied by the references’ actual values. Hence, unit 87 should reduce all the inputs while maintaining all the outputs constant to be considered efficient in model A.

| **Units** | **MD/**  **10Beds** | **Nur/**  **10Beds** | **NurTec/**  **10Beds** | **Physio/**  **10Beds** | **SMR** | **SRU** | **% Ref** |
| --- | --- | --- | --- | --- | --- | --- | --- |
| **Non-efficient** | | | | | |  |  |
| 87 | 0.78 | 1.6 | 5.88 | 0.8 | 1.22 | 1.09 |  |
| **References** | | | | | | | |
| 18 | 1.24 | 3.18 | 14.21 | 1.13 | 0.64 | 0.58 | 6.33% |
| 51 | 0.86 | 1.35 | 3.71 | 0.86 | 0.58 | 1.31 | 6.01% |
| 80 | 0.62 | 0.62 | 2.81 | 0 | 1.41 | 1.15 | 87.66% |
| **Targets** |  |  |  |  |  |  |  |
| 87 | 0.67 | 0.83 | 3.59 | 0.12 | 1.22 | 1.09 |  |

MD/10Beds – number of physicians per 10 beds; Nur/10Beds – number of nurses per 10 beds; NurTec/10Beds – number of nursing technicians per 10 beds; Physio/10Beds – number of physiotherapists per 10 beds; SMR – Standardized Mortality Ratio; SRU – Standardized Resource Use; % Ref – percentage of reference of each reference unit.
