# Supplementary figures and images for: Using data envelopment analysis to perform benchmarking in intensive care units
Source: PLoS One. 2021 Nov 18;16(11):e0260025. doi: 10.1371/journal.pone.0260025 (PMC8601512; doi:10.1371/journal.pone.0260025)

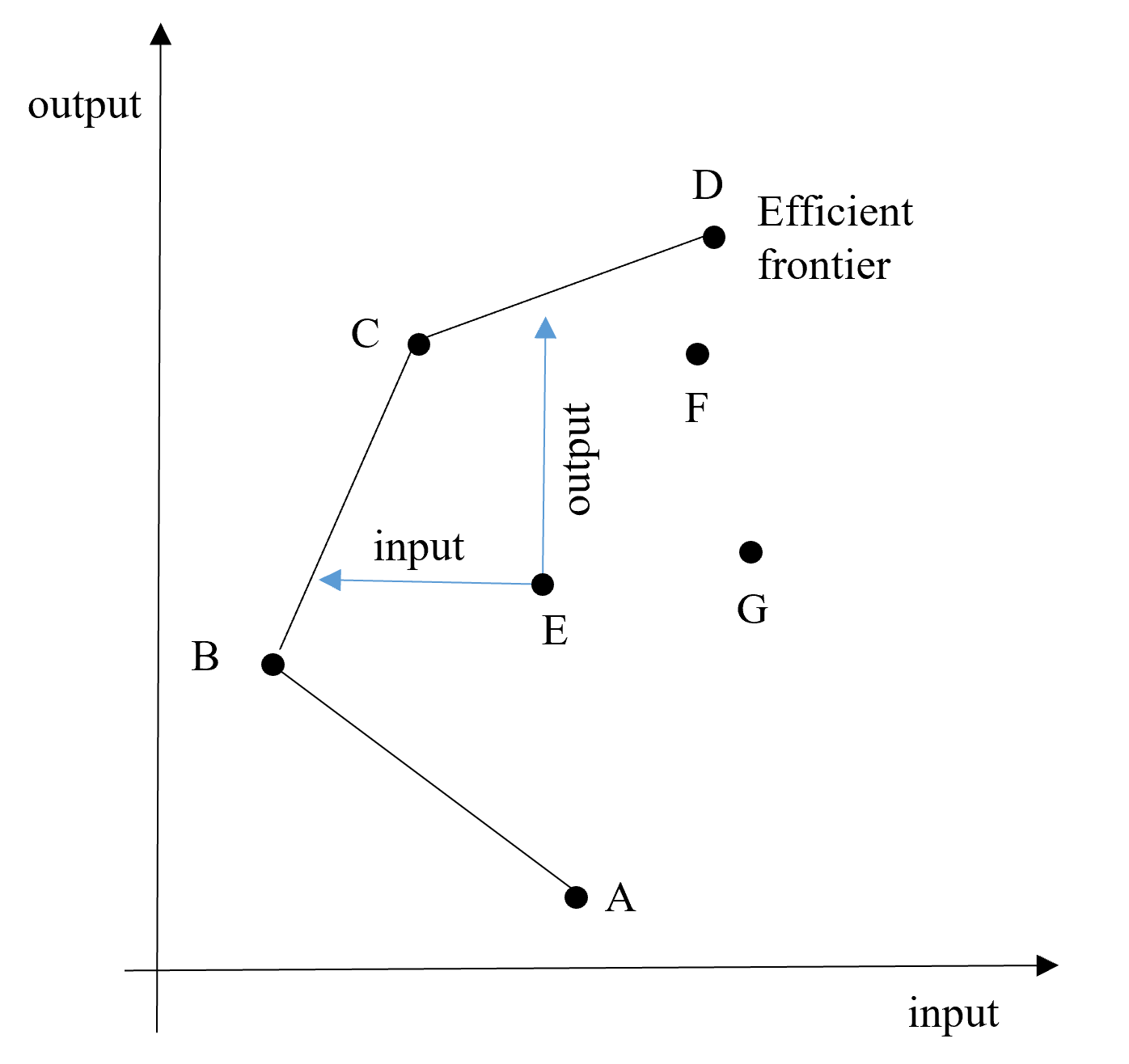

Supplement: S1 Fig — (TIF) [file pone.0260025.s007.tif]
